# Thank you to all our manuscript reviewers in 2014

**DOI:** 10.1186/s13053-015-0029-y

**Published:** 2015-03-12

**Authors:** Jan Lubinski, Rodney J Scott, Rolf Sijmons, Katie Bayliss

**Affiliations:** 1 Pomeranian Medical University, Department of Genetics and Pathomorphology, Szczecin, Poland; 2 Discipline of Medical Genetics, Faculty of Health, University of Newcastle and Hunter Medical Research Institute, New South Wales, Australia; 3 Department of Genetics, University Medical Center Groningen, Groningen, The Netherlands; 4 BioMed Central, 236 Gray’s Inn Road, London, WC1X 8HB United Kingdom

## Abstract

The editors of *Hereditary Cancer in Clinical Practice* would like to thank all our reviewers who have contributed to the journal in 2014.

Without the participation of skilful reviewers, no academic journal could succeed, and we are grateful to the committed individuals who have given their time and expertise to the peer review of manuscripts for *Hereditary Cancer in Clinical Practice*. We look forward to your continued support in 2015.

**Annie Anderson**

UK

**Stefan Aretz**

Germany

**Patricia Ashton-Prolla**

Brazil

**Kelly Avery-Kiejda**

Australia

**Cecelia Bellcross**

USA

**Lev Berstein**

Russia

**Morgan Butrick**

USA

**Daniele Calvano-Mendes**

Brazil

**Cezary Cybulski**

Poland

**Lijun Di**

China

**Katarzyna Durda**

Poland

**Maurizio Genuardi**

Italy

**Ad Geurts van Kessel**

Netherlands

**Grey Giddins**

UK

**Eli Marie Grindedal**

Norway

**Jacek Gronwald**

Poland

**Eliete Guerra**

Brazil

**Parry Guilford**

New Zealand

**Shirley Hodgson**

UK

**Veronica Höiom**

Sweden

**Nicoline Hoogerbrugge**

Netherlands

**Gill Hubbard**

UK

**Kay Huebner**

USA

**Evgeny Imyanitov**

Russia

**Arvids Irmejs**

Latvia

**Anna Jakubowska**

Poland

**Anita Kinney**

USA

**Marcin Lener**

Poland

**Noralane Lindor**

USA

**Jose Lozano**

Spain

**Henry Lynch**

USA

**Finlay Macrae**

Australia

**Diptasri Mandal**

USA

**Robert McWilliams**

USA

**Lawrence Merritt**

USA

**Steven Narod**

Canada

**Katherine Nathanson**

USA

**Maartje Nielsen**

Netherlands

**Carla Oliveira**

Portugal

**Domenico Palli**

Italy

**Edenir Palmero**

Brazil

**Helle Vendel Petersen**

Denmark

**Carmen Radecki Breitkopf**

USA

**Thangarajan Rajkumar**

India

**Adelita Ranchor**

Australia

**Iradj Sobhani**

France

**Melissa Southey**

Australia

**Allan Spigelman**

Australia

**Bente Talseth-Palmer**

Australia

**Bryony Thompson**

Australia

**Sergio Toledo**

Brazil

**Katarzyna Tutlewska**

Poland

**Alexandre Rezende Vieira**

USA

**Rinse Weersma**

Netherlands

**Smith Wolf**

Pakistan

**Michelle Wong-Brown**

Australia

**Yuan Yuan**

China

